# A Novel Approach for Development of Intraocular Biodegradable Ranibizumab Implant: A Solution for Stability of Protein Activity

**DOI:** 10.34172/apb.2021.072

**Published:** 2020-10-20

**Authors:** Fatemeh Dadgar Pakdel, Ahmad Mirshahi, Payam Zahedi, Kazem Mohammad, Farkhondeh Hemmati, Javad Dadgar Pakdel, Mohammad Hossein Nicknam, Farid Abedin Dorkoosh

**Affiliations:** ^1^Department of Immunology, School of Medicine, Tehran University of Medical Sciences, Tehran, Iran.; ^2^Department of Pharmaceutics, Faculty of Pharmacy, Tehran University of Medical Sciences, Tehran, Iran.; ^3^Department of Ophthalmology, School of Medicine, Tehran University of Medical Sciences, Tehran, Iran.; ^4^Department of Polymer, School of Chemical Engineering, College of Engineering, University of Tehran, P. O. Box: 11155-4563, Tehran, Iran.; ^5^Epidemiology and Biostatistics Department, School of Public Health, Tehran University of Medical Sciences, Tehran, Iran.; ^6^Caspian Faculty of Engineering, College of Engineering, University of Tehran, P.O.BOX 43841-119, Gilan, Iran.; ^7^Department of Pharmaceutical Biotechnology, Pasteur Institute, Tehran, Iran.; ^8^Molecular Immunology Research Center, Tehran University of Medical Sciences, Tehran, Iran.; ^9^Medical Biomaterial Research Centre (MBRC), Tehran University of Medical Sciences, Tehran, Iran.

**Keywords:** Ranibizumab, Implant, VEGF-A, Extrusion method, Sustained release

## Abstract

***Purpose:*** Ranibizumab is a monoclonal antibody fragment, targeting all isoforms of vascular endothelial growth factor A (VEGF-A), a protein involved in angiogenesis. It is used to treat age-related macular degeneration (AMD), retinal vein occlusion (RVO), and diabetic macular edema (DME), which are associated with blindness worldwide. However, proper treatment can decrease the loss of vision in about 90% of patients. Because of poor drug uptake in topical therapy and several adverse side effects of systemic irregularities and intravitreal injections, sustained-release drug delivery systems are more suitable for treatment. However, there are many challenges in the development of these systems due to the loss of protein activities.

***Methods:*** After drug complexation by the ion pairing method and preparation of a polymeric implant, containing the drug, the characteristics of the complexes were examined by Fourier-transform infrared spectroscopy and circular dichroism spectroscopy. The stability of antibody activity and biocompatibility of the released drug from the implant were assessed by bioassays and MTT assay, respectively. Finally, the release kinetics were investigated.

***Results:*** The bioassays showed the higher activity of the drug complex, compared to the free form, besides good biocompatibility *in vitro*. Also, the release data confirmed sustained and controlled release characteristics for the prepared implant.

***Conclusion:*** In this study, for the first time, we proposed a method for developing a sustained-release intraocular implant, consisting of ranibizumab by the heating method. This method allows for the industrial production of ranibizumab by extrusion and eliminates the complications related to reservoir systems.

## Introduction

Considering the anatomical structure and physiological function of the eyes and eye tissues, design of ophthalmic drugs has always been a major challenge. Eye drops and ointments are conventional medications for the treatment of ophthalmic diseases. The use of these drugs minimizes drug delivery to the posterior region of the eye. Therefore, they are commonly used to treat anterior ocular diseases. Meanwhile, posterior-segment eye diseases, such as diabetic retinopathy, macular edema, and macular degeneration, cause visual impairments in developed countries. Treatment of neovascularization in the choroid and posterior areas is one of the major problems, although many efforts are being currently made to deliver drugs to these posterior regions.^1-3^

In topical drug administrations, poor drug access is related to barriers in the eyes and rapid release of drugs, resulting in a series of adverse side effects due to systemic drug absorption. In systemic administrations, drug access to different areas of the eye is low, owing to the presence of a blood-ocular barrier.^1-3^ On the other hand, intravitreal injection is the most appropriate method for delivering medications to the posterior segments of the eye, ensuring an effective therapeutic concentration of the drug. Despite the popularity of this method, sequential injections are essential due to the limited half-life of many drugs. Therefore, serious eye complications may occur, such as intravitreal bleeding, endophthalmitis, retinal detachment, and cataract development.^4-6^

Intraocular polymeric implants are among other delivery systems to the posterior segment of the eye.^7^ They can bypass the ocular barrier, eliminate the adverse side effects of repeated intravitreal administrations, and reduce complications associated with the lower amount of drug in the treatment process.^7-9^ These systems can also extend the duration of drug release using suitable polymers. However, the development of such implants, containing protein-based drugs, has many problems, such as instability of the structure, loss of activity, and aggregation of drug during formulations. Recently, several studies have examined the production of non-biodegradable systems, including port delivery system with ranibizumab, to minimize the problems of biodegradable systems.^10,11^

On the other hand, special attention has been paid to anti-vascular endothelial growth factor (VEGF) drugs, such as ranibizumab monoclonal antibody, for the treatment of ocular diseases, including diabetes-induced retinopathy and age-related macular degeneration (AMD) due to angiogenesis. Ranibizumab is the best treatment option for these patients to prevent the progression of disease. However, monthly injection into the vitreous of the patient’s eye is an invasive procedure with many side effects.^11-17^ Therefore, production of drug-containing implants can reduce these side effects and decrease the ongoing hospitalization costs for patients.

In the present study, we developed a polymer-based implant, containing ranibizumab (Lucentis), and optimized it to achieve optimal drug release. Also, a novel approach was proposed to design a sustained-release drug delivery system with ranibizumab, using the melting method. In this approach, the antibody activity was maintained high, and this technique can be used to produce biodegradable implants based on poly (3-hydroxybutyric acid-co-3-hydroxyvaleric acid) (PHBV) containing the protein on an industrial scale, using a twin screw extruder.

## Materials and Methods

### Materials

PHBV with 3 wt% polyhydroxyvalerate (PHV) was supplied by Tianan Biologic Materials Co. Ltd. (Ningbo, China). Polyethylene glycol 600 (PEG600) and M199, as the cell culture media, were purchased from Sigma-Aldrich (St. Louis, USA). Dextran sulfate sodium (DS) from *Leuconostoc* spp. with a relative molecular mass (M_r_) of 5000, as well as hydrochloric acid (HCl), was provided by Merck (Germany). Ranibizumab (Lucentis^®^, 10 mg/mL) was obtained from Genentech Inc. (CA, USA). Also, a microBCA^^TM^^ Protein Assay Kit and phosphate-buffered saline (PBS) tablets were purchased from BioBasic (Canada). The human umbilical vein endothelial cells (HUVECs) and fibroblast cell line were also supplied by Pasteur Institute of Iran. Finally, fetal bovine serum (FBS), penicillin, and streptomycin were provided by Invitrogen (Life Technologies, UK), and anti‐Ki67 antibody was supplied by Abcam (LA, USA).

### Preparation of antigen-binding fragment complexes from the mixture of ranibizumab and DS

The antigen-binding fragment complexes (Fab.com) were prepared from the mixture of ranibizumab (Fab) and DS, based on the hydrophobic ion pairing (HIP) technique. To select the optimal pH in this process, ranibizumab: DS molar ratio was adjusted to 2.5 and the binding efficiency percentage was investigated at different acidic pH values (4, 4.5, 5, and 5.5, adjusted by 0.1 N HCl). In this regard, the solution containing DS and ranibizumab was vortexed at the mentioned molar ratio to form the HIP complex. Subsequently, it was centrifuged at 14 000 rpm for 15 minutes to separate the supernatant, including the non-complexed antibody fragments. Finally, the samples were washed three times and lyophilized. The fragments were measured, based on the binding efficiency criterion of the HIP reaction, using a Micro BCA^TM^ Protein Assay Kit.

After determining the proper pH at a specific DS: ranibizumab molar ratio, the effects of different molar ratios (0.63, 1.25, 2.5, and 5) on the binding efficiency (%) were evaluated at pH 4. The procedures for mixture preparation and fragment separation in this step were similar to optimal pH determination. The Fab complexation efficiency (CE%) was calculated according to equation (1):

*CE* (%) = *M*_i_− *M*_f_/(*M*_i_× 100%) Eq. (1)

Where *M*_i_ and *M*_f_ represent the initial amount of ranibizumab added to the reaction and the free amount of ranibizumab in the supernatant, respectively.

### Characterization of Fab.com

#### Aqueous solubility

To determine the aqueous solubility, 0.1 mg of the resulting complex was added to 1 mL of deionized water and placed in a shaker bath at room temperature. After 24 hours, the solution was centrifuged at 14 000 rpm for ten minutes. The obtained supernatant was filtered through a 0.45-μm syringe filter, and the Fab concentration was measured by a Micro BCA assay three times.^18^

#### Fab.com dissociation in water, PBS, and rabbit vitreous fluid

To determine the nature of interactions between the ranibizumab antibody (Fab) and DS, the dissociation properties of the HIP complex were investigated in three media at room temperature. Briefly, 0.1 mg of the resulting complex was added to 1 mL of PBS (10 mM). After vortexing, the solution was kept at room temperature for 6 hours, and then, centrifuged at 14 000 rpm for ten minutes. Next, the concentration of the collected supernatant was measured. The same method was used for the supernatant extraction from deionized water and vitreous fluid environments.

#### Functional groups of DS, ranibizumab, and Fab.com

To investigate the formed bonds between DS and ranibizumab after the chemical reaction, Fourier-transform infrared spectroscopy (FTIR; Tensor 27, Bruker, USA) was performed at wavelengths of 500–4000 cm^−1^. Also, this method was used to assess the drug-polymer interactions.

#### Stability studies of Fab.com structure

To evaluate the secondary structure stability of Fab.com before and after complexation, circular dichroism (CD) was performed in a J-810 spectropolarimeter (Jasco Inc., USA) with a cylindrical quartz cuvette (0.1 cm in length). After dissociation of ranibizumab from DS in PBS (10 mM), the Fab protein was desalted and dialyzed against water, and then, scanned at wavelengths of 190-350 nm at a speed of 200 nm/min.

### Preparation of PHBV implants containing Fab and Fab.com

To prepare two rod-shaped implants containing Fab and Fab.com (Fab.com), PHBV as a base polymer and PEG as a pore former/plasticizer were first mixed at a weight ratio of 1:1 for five minutes at 160°C. After cooling, the sample was re-heated up to 100°C, and the drugs were added to the molten polymer; the mixture was stirred for five minutes, followed by casting on ice.

### Assessment of Fab.com release of PHBV implant

#### Enzyme-linked immunosorbent assay (ELISA)

To measure the concentration of Fab, an ELISA assay was carried out. First, polystyrene plates were coated with 50 µL of VEGF165 recombinant protein stock and incubated overnight at 4°C. Next, each well was washed three times with 0.05% Tween-20 in PBS, and then, the wells were blocked with 5% bovine serum albumin (BSA) for two hours at 4°C. After washing three times, the samples were incubated for one hour at room temperature. Then, a horseradish peroxidase (HRP)-conjugated anti-human antibody, diluted in PBS (1:5000), was added to each well and incubated for one hour at 37°C. After washing three times, a 3,3′,5,5′-tetramethylbenzidine (TMB) substrate was added, and the plate was incubated for 20 minutes in a dark room. Finally, the reaction was terminated by using 1 M sulfuric acid, and absorbance was read at 430 nm with an ELISA reader (DANA-3200, Garni Medical Engineering Co., Iran).

#### Cell culture

The HUVECs were cultured in the M199 medium, supplemented with 20% FBS and 3% penicillin/streptomycin. After incubation at 37°C in a humidified atmosphere (5% CO_2_), the cells were seeded on plates and allowed to grow to at least 80% confluency for endothelial cell tube formation and cell proliferation assays. To culture the fibroblast cells (L929), the Dulbecco’s Modified Eagle Medium (DMEM), supplemented with 1% penicillin/streptomycin and 1% FBS, was used.

#### Endothelial cell tube formation

A Matrigel-based tube formation assay was performed on HUVECs in the presence of Fab and Fab.com, released from the prepared implants. Briefly, a Corning Matrigel matrix, as the basement membrane, was thawed at 4°C overnight, mixed with the M199 medium, and poured in a 96-well plate. The membrane was incubated at 37°C for 30 minutes to allow for solidification. After harvesting the cells by trypsin, they were seeded at 10^4^ cells/well, using the M199 medium, supplemented with 1% FBS for 24-48 hours. The cell tube formation was assessed by inverted light microscopy (BX-51, Olympus, Japan). Also, to evaluate tubulogenesis, a colony scoring system was used.^19,20^ The tube length of capillary-like structures was investigated in ImageJ version 1.36.

#### Cell proliferation assay

To compare the proliferative potential of HUVECs, induced by 678 ng/mL of anti-VEGF (standard ranibizumab, Fab.com, and Fab), two methods were employed for the cell proliferation assay. First, the cells were stained by Ki67.^21^ For this purpose, they were washed with PBS, blocked with BSA, and then incubated with an anti‐Ki67 antibody for two hours at 4°C. Next, the treated cells were incubated with Texas red goat anti-rabbit IgG (diluted 1:1000 in PBS containing 1% BSA) and washed with PBS for one hour at room temperature. They were then counterstained with 4′,6-diamidino-2-phenylindole (DAPI). Finally, the stained cells were analyzed, using a multi-band fluorescent filter to quantify the percentage of Ki‐67-positive cells.

In the second method, 10×10^4^ HUVECs were seeded on a 24-well culture plate in the presence of M199 medium, supplemented with 678 ng/mL of anti-VEGF. The effect of anti-VEGF on the cell proliferative rate was evaluated, using a growth curve analysis. The cell count was determined daily over 128 hours, using a cell counter (MEK-6450K Celltac Alpha, Nihon Kohden, Germany). The cell count was finally plotted against the culture time to plot the growth curve.

### MTT toxicity assay

To evaluate the non-cytotoxic properties of the compounds released from the polymeric matrix implants containing Fab and Fab.com, the fibroblast cell viability was determined using MTT assay after 32 days. For this purpose, Fab and Fab.com were added to the cells after 12 hours. Next, the cells were washed with PBS after 48 hours, and the MTT reagent was added to the cells. The mixtures were then incubated at 37°C for five hours, and the MTT medium was replaced with dimethyl sulfoxide (DMSO) in each well. The quantity was immediately measured with an ELISA reader at 570 nm.

### Field emission-scanning electron microscopy (FE-SEM)

The FE-SEM analysis, using a MIRA III microscope (TESCAN Co., Czech Republic) at 20 kX magnification, was implemented to determine the morphology of the Fab.com-loaded implant before and after PBS immersion during 32 days. Before imaging, the surfaces were coated with a thin layer of gold.

### Release kinetics of the prepared rod-shaped implant

The drug release from the prepared implant in the PBS medium (pH=7.4) was measured by ELISA assays after 48, 96, 192, 384, and 768 hours at 37˚C. The drug release kinetic models such as zero-order, first-order, Higuchi and Hixson-Crowell were investigated and the model with the best fit to the experimental data with the highest regression coefficient (R^2^) was selected.

### Statistical analysis

Data are presented as mean±SD. After evaluating the homogeneity of variance and normal distribution of data, statistical analysis was performed, using one-way analysis of variance (ANOVA) for more than two groups and Tukey’s post hoc test. Statistical analysis was performed in GraphPad Prism version 8.0.2. *P* value ≤0.05 was considered statistically significant.

## Results and Discussion

### Determination of optimal pH and molar ratio for Fab.com formation

**Figure 1:**
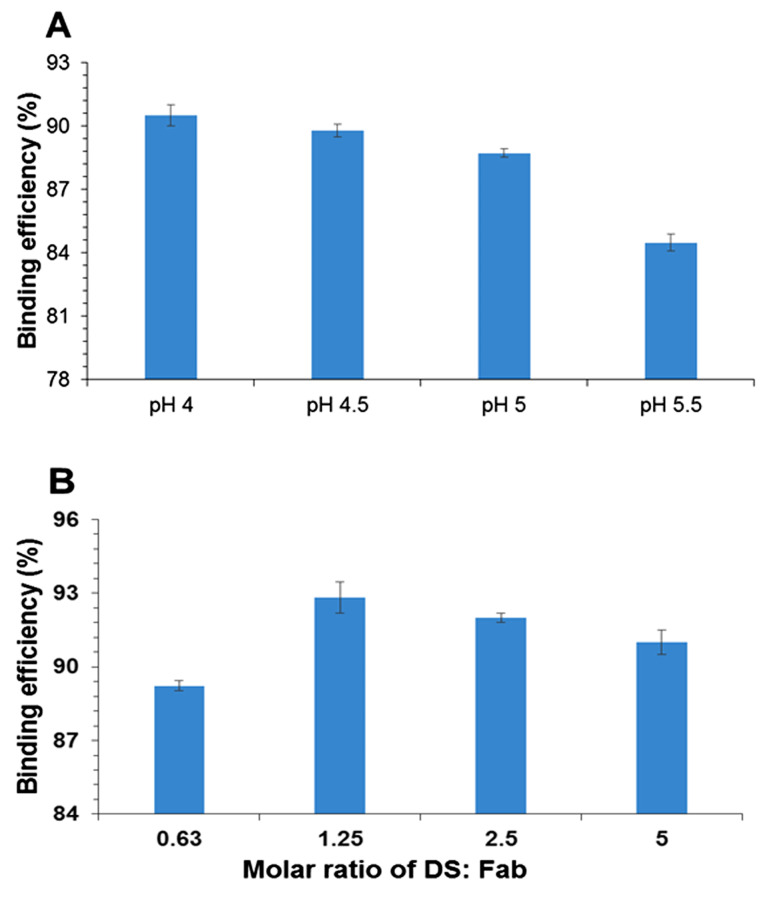
(A) Effect of pH on % binding efficiency of ranibizumab (Fab) and dextran sulfate sodium (DS); and (B) effect of molar ratio on % binding efficiency of Fab with DS as ion-pairing agent (Values were represented as mean ± SD, n = 3).

The HIP complex of Fab and DS (as the ion pairing agent) was prepared, and its binding efficiency percentage was measured. Based on the results of previous studies, the pK_a_ of sulfate group in DS was < 2; therefore, this molecule had a negative charge above pH 2.^22^ The effects of four different pH ranges and molar ratios of DS to Fab were evaluated to achieve maximum binding. A molar ratio of 1.25 at pH 4 was selected for drug complexation (Figure 1A and 1B). The isoelectric pH of ranibizumab was 8.8, and the surface charge of the protein at pH < 4 was positive; therefore, ionic interactions occurred between the negatively charged DS and the positively charged Fab.^23^ The results showed that by decreasing pH, the surface charge of protein increased. As shown in Figure 1A, the binding efficiency percentage was increased by decreasing pH.

The results showed an increase in the binding efficiency of Fab with an increment in the molar ratio up to 1.25 (Figure 1B). The maximum percentage of binding efficiency of Fab to DS was observed when the surface charge ratio of DS to protein was around 1:1.^22^ At a molar ratio of 1.25, the surface charge of protein and DS was approximately equal, and maximum binding was observed. Regarding the HIP mechanism, DS was selected as an ion pairing agent because of its close molecular weight to the drug.^22^ Many researchers have selected other ion pairing agents and protein drugs with significant differences in their molecular weight, resulting in a high molar mass ratio.^21^ Therefore, DS was selected in the present study.

### Characterization of Fab.com

#### Dissociation of ranibizumab from the HIP complex and aqueous solubility

**Figure 2:**
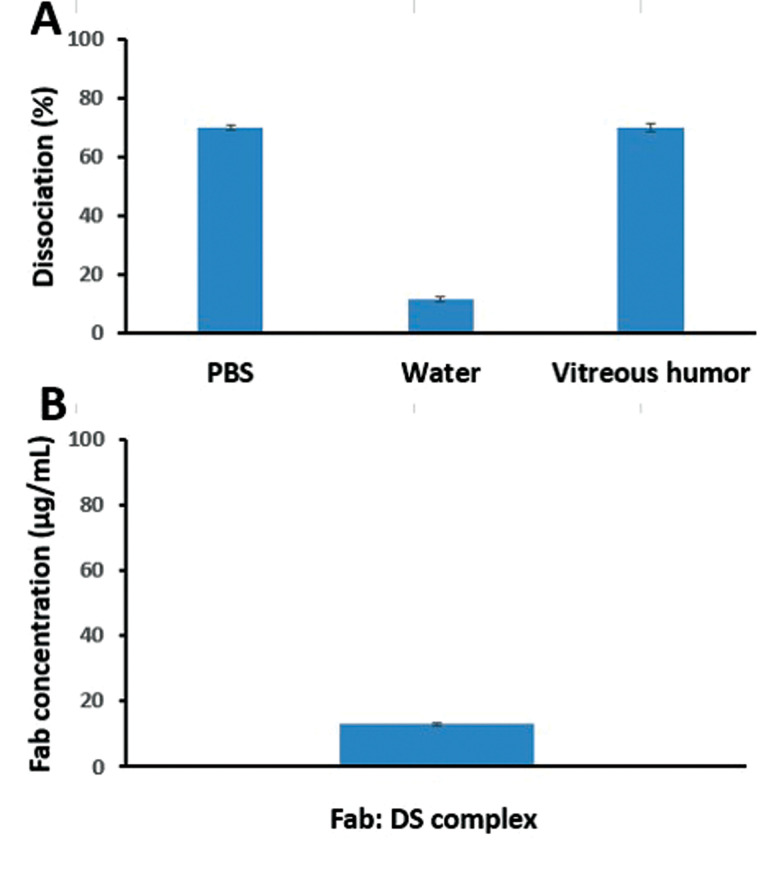
(A) Dissociation of ranibizumab (Fab): DS HIP complex (Fab.com) in phosphate buffered saline (PBS), water, and vitreous humor at room temperature after 6 h; and (B) aqueous solubility of Fab.com after 24 h (Values were represented as mean ± SD, n = 3).

The dissociation of HIP complex was evaluated in the presence of PBS (10 Mm, pH=7.4), vitreous fluid (from rabbit), and deionized water at room temperature (Figure 2A). The dissociation percentage of the complex in the presence of PBS and vitreous fluid was similar. As mentioned in other previous studies, ionic interactions are important factors in the formation of complexes and are eliminated in environments with a higher ionic strength. The dissociation tests indicated the importance of these interactions in complexation; this feature can be used for dissociating the drug *in vivo*.^21^ In the present study, the dissociation percentage of the PBS medium was about 70%, which might represent the possible presence of hydrophobic bonds between hydrophobic amino acids and hydrophobic regions of DS.^21^ On the other hand, the dissociation percentage in the aqueous medium and aqueous solubility were approximately 10%, indicating stable ionic interactions of HIP complexes in the medium with a very low ionic strength.^22,24^ The presence of such ionic interactions in the formation of drug complexes has been also confirmed.^21^ Overall, the results of the present study confirmed the results of some previous research.^21,23^

#### HIP mechanism in Fab.com by functional group formation

**Figure 3:**
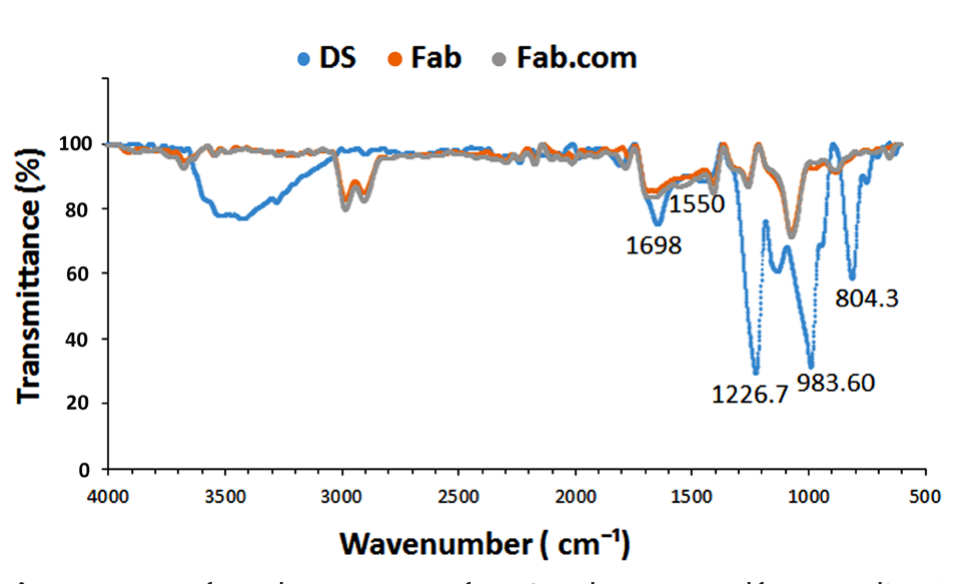
Infrared spectra of DS (dextran sulfate sodium), ranibizumab (Fab), and Fab: DS complex (Fab.com).

To investigate the nature of interactions between the sulfate groups of DS and amino acid groups of ranibizumab, their characteristic peaks are shown in Figure 3. The observed peaks at 804.3 cm^-1^, 983.6 cm^-1^, and 1226.7 cm^-1^ corresponded to S―O―S vibration, symmetric SOO― stretching vibration, and asymmetric SOO― stretching vibration, respectively. Generally, during HIP reactions, there is an attenuation or shift related to the sulfate groups of DS because of the formation of ionic interactions between the functional groups.^18^ Accordingly, the IR peaks for sulfate groups in DS were attenuated in the present study.

Moreover, the stability of secondary protein structures was examined in this study. Amide I and II bands are generally two major functional groups for characterizing the secondary structure of proteins.^25^ In the infrared spectrum of ranibizumab antibody, the peaks pertaining to amide I (1698 cm^-1^) and amide II (1550 cm^-1^) bands of Fab.com and Fab were similar and remained constant, indicating the stability of the secondary structure of antibody before and after complexation.^21^

#### Secondary structural changes of Fab after complexation with DS

**Figure 4:**
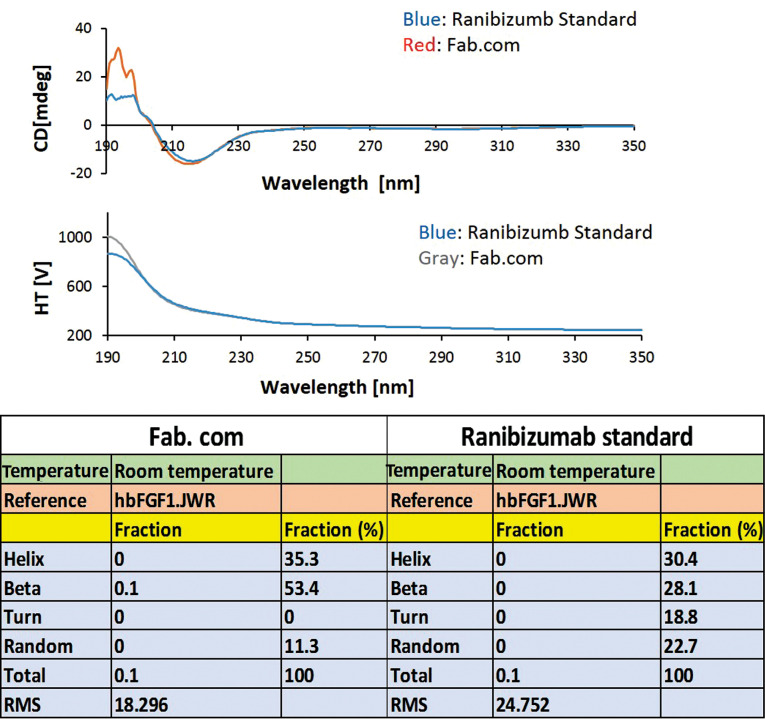
Circular dichroism spectroscopy (CD) test (ranibizumab standard, and ranibizumab (Fab): DS complex (Fab.com).

To investigate the conformational changes due to complexation with DS, a CD test was performed (Figure 4). Generally, the secondary structural elements and their fractions can be assessed by CD test.^26^ According to the literature, formation of a protein complex with DS can induce structural changes in the protein and lead to the induction of β-sheets in the protein structure; these structural changes are different at various pH values.^27^ The present results showed an increase in the percentage of β-sheets and a decrease in the percentage of β-turns and random coils after drug complexation. The percentage of α-helix structure in the drug complex increased slightly. Although there were changes in the secondary structure of the antibody, it was unclear whether these structural changes had a negative effect on the drug activity. Therefore, antibody activity was measured using complementary tests, including the tube formation assay and proliferation test.

### Characterization of released Fab and Fab.com from the implant bybioassay tests

#### Tube formation test (Matrigel assay)

The effects of released Fab and Fab.com from the PHBV implant were evaluated on tubulogenesis by evaluating the tube length and colony score. Two control samples were considered, that is, HUVECs without treatment and HUVECs treated with standard ranibizumab (control and standard, respectively). According to the literature, 25 colonies were examined per well, and a score of 0-4 was assigned to each colony as follows: aggregation with no sprouting (0); colony sprouting (1); colony sprouting with arborization (2); arborization with anastomosis (3); and complex network (4).^19,20^ The total score was calculated, based on the total score of 25 colonies per well (maximum score=100). To evaluate the length of tubes, the average length of 50 tubes was calculated in ten serial microscopic fields. The length of formed tubes was measured in micrometers using ImageJ software.

**Figure 5:**
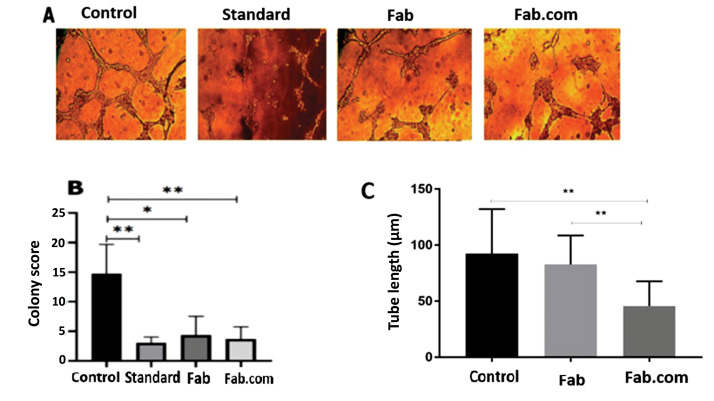
Tube formation assay: (A) the effect of ranibizumab standard, ranibizumab (Fab), and Fab: DS (dextran sulfate sodium) complex (Fab.com) on tubulogenesis in human umbilical vein endothelial cells (HUVECs); (B) colony score; and (C) tube length. Values were represented as mean ± SD, n = 3. Three independent tests were done in triplicate. * *P*  <  0.05, ** *P*  <  0.01, and *** *P*  <  0.001. One-way ANOVA with Tukey post hoc test.

Ranibizumab (Lucentis^®^) is a humanized monoclonal antibody fragment (Fab) of IgG1 against human VEGF-A. VEGF-A glycoprotein is the main factor in angiogenesis.^28^ As shown in Figures 5A-5C, the colony score and tubular length decreased in the standard, non-complex, and complex forms of the drug, compared to the control, owing to the anti-angiogenic effect of this drug. The activity of Fab.com in comparison with Fab was largely maintained after the heating process. In a study conducted in 2019 on the thermal stability of IgG1 antibody, it was found that almost half of the binding activity of the antibody decreased after two minutes at 90°C, and Fab (e.g., ranibizumab) was very sensitive to heat treatment.^26,29,30^ The present results also indicated that half of the antibody activity decreased in *in vitro* angiogenesis of HUVECs after about five minutes of heating at 100°C, whereas Fab complexation led to higher thermal stability.

#### Cell proliferation assay

**Figure 6:**
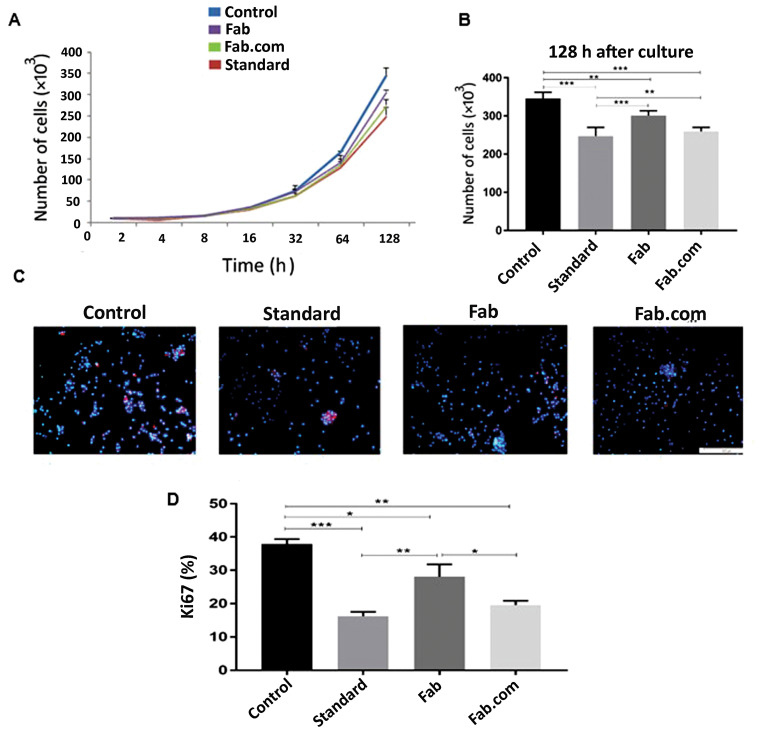
The cell proliferation test: (A, B) the results of human umbilical vein endothelial cells (HUVECs) proliferation by cell counting; (C) the results of Ki-67 immunostaining; and (D) Ki-67 expression percent in HUVECs. (Control: cells with no drug treatment, Standard: cells treated with ranbizumab standard, Fab: ranibizumab, and Fab.com: Fab: dextran sulfate sodium (DS). Values are represented as mean ± SD (n = 3). Three independent tests were done in triplicate. * *P*  <  0.05, ** *P*  <  0.01, *** *P*  <  0.001. One-way ANOVA with Tukey post hoc test.

To compare the stability of antibody activity between Fab and Fab.com, a cell proliferation assay was conducted (Figures 6A-6D). Figure 6A and Figure 6B showed a significant difference in cell proliferation after 128 hours, and the level of cell proliferation in the Fab.com group showed a more significant decrease than the Fab and other groups. On the other hand, Ki-67 is a nuclear antigen, involved in cell proliferation. This molecule can be used as a marker of cell proliferation due to its expression during the cell cycle and cell division, while it is absent in quiescent cells.^31^ In immunostaining, as shown in Figure 6C and Figure 6D, cell proliferation by Ki-67 was seen in red, while nuclei stained by DAPI (DNA stain) were blue.

According to the literature, given the effect of ranibizumab antibody, the ratio of ki-67-positive cells to the total DAPI-positive cells decreases. This ratio is lower in complexed drugs than uncomplexed drugs, indicating that drug activity is maintained to a greater extent. Ki-67 is expressed in cells during all stages of cell proliferation, including G1, S, G2, and mitosis. This antigen is the best marker used to identify cells in the proliferation phase and is never expressed in the G0 phase.^32^ Compared to its free form, the complex form of the drug shows higher activity stability, and consequently, a higher anti-proliferative effect on HUVECs.

In the present study, the activity of the complex drug decreased slightly, compared to the standard. Overall, our results showed that drug complexation by the mentioned protocol provides a resistant form of drug to the heating process, which is required for the preparation of a biodegradable implant. The results of previous studies on various proteins have shown that conjugation of proteins with carbohydrates, including DS, leads to an increase in protein resistance to high temperatures, and their addition to protein solutions can improve their thermal stability. This thermal stabilizing effect of carbohydrates depends on their concentration and type, as well as the pH of the environment.^33^

### MTT assay

**Figure 7:**
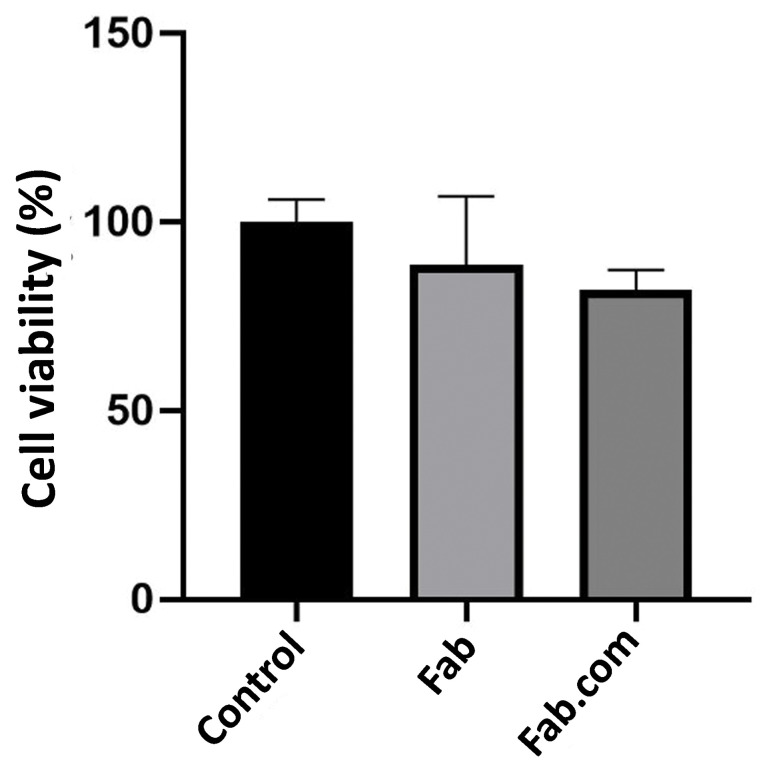
The MTT assay: ranibizumab (Fab), Fab: dextran sulfate sodium (DS) complex (Fab.com), and L929 fibroblast cells with no treatment (control).

The released media from two implants, containing Fab and Fab.com with DS, were investigated using MTT assay after 32 days. The percentage of cell viability was above 80% in both implants; therefore, there was no toxicity related to the released components after 32 days (Figure 7). The United States Food and Drug Administration (FDA) approved 0.3 mg of ranibizumab (Lucentis^®^) for treating all forms of diabetic retinopathy monthly. The intravitreal injection of 0.5 mg of Lucentis (0.05 mL) is recommended once a month for patients with AMD and retinal vein occlusion. Based on the results, the amount of drug released after one month did not have any toxic effects on the cells. Also, PEG is an FDA-approved non-ionic hydrophilic polymer, used in many pharmaceutical compounds, such as liquid PEGs (PEG 200-600) as oral liquids and parenterals.^34,35^

### Morphological studies of PHBV implant containing Fab.com

**Figure 8:**
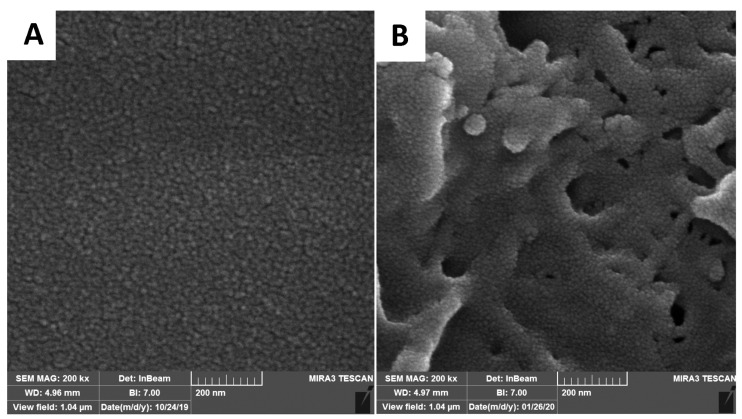
Field-emission scanning electron microscopic (FE-SEM) images of the prepared poly (3-hydroxybutyrate-co-3-hydroxyvalerate) (PHBV) implant containing ranibizumab (Fab): dextran sulfate sodium (DS) complex (Fab.com): (A) before the drug release, and (B) after the drug release during 32 days (The scale bar is 0.2 μm).

Relative to the sample surface, almost uniform and homogenous polymer and drug were obtained before the Fab.com release (Figure 8A). At the end of day 32 of drug release in PBS, the PHBV/PEG polymer implant was dried at room temperature, and its morphology was re-evaluated. Considering the presence of PEG600 as the pore-forming agent in the prepared polymeric implant, the implant surface showed a porous structure (Figure 8B). After one month, the implant containing the drug showed a porous surface in the micrograph. The results of previous studies have also shown that the PHBV scaffold has a porous structure in PBS medium after 4 weeks, and the presence of pore-forming agents, such as PEG, creates more pores in the structure, which is in line with the results of the current study.^33,36^

### Investigation of drug-polymer interactions by FTIR spectral analysis

**Figure 9:**
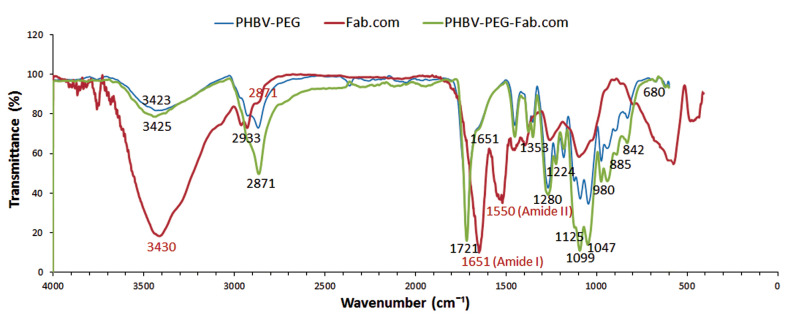
Infrared spectra of poly (3-hydroxybutyrate-co-3-hydroxyvalerate) (PHBV)-poly (ethylene glycol) (PEG 600), ranibizumab (Fab): dextran sulfate sodium (DS) complex (Fab.com), and PHBV-PEG 600-Fab.com.

The FTIR analysis was performed to assess the drug-polymer interactions. The important peaks related to the drug were identified and compared with the peaks related to the heated mixture of ranibizumab and polymer. Because of the importance of amide I (at 1651 cm^-1^ corresponding to C=O bond) and amide II (at 1550 cm^-1^ corresponding to N―H bond) bands in the secondary structure of protein, they were evaluated in the FTIR spectra of ranibizumab antibody and the drug-polymer mixture. The FTIR spectra of PHBV-PEG600 mixture is shown in Figure 9, which represents the important peaks of PHBV polymer: 3425 cm^-1^ (―OH group); 2933 cm^-1^ and 2871 cm^-1^ (―CH_2_ and ―CH_3_ groups) 1721 cm^-1^ (C=O group), 1459 cm^-1^ (―CH_2_ group); 1358 cm^-1^ (―CH_3_ group); 1000-1300 cm^-1^ (C–O–C stretching mode); and 980 cm^-1^ (―CH_2_ group). It seems that the fingerprint bands at 1000-1300 cm^-1^ and the peak at 980 cm^-1^ are major bands in PHBV identification.^37,38^ The peaks at 2933 cm^-1^ (―CH2 group) and 1000-1300 cm^-1^ could be attributed to both PHBV and PEG 600.^39^

Because of the heating process, the intensity of amide I band in the drug-polymer mixture decreased at 1651 cm^-1^. On the other hand, the amide II band at 1550 cm^-1^ was disappeared. The lack of amide II band at this position and the decreased intensity of amide I band are probably related to structural changes in protein as a result of heating; the results of bioassays in the present study confirm these results. However, the results of other studies show a shift in amide I band after the heating process, which indicates the formation of intramolecular β-sheets, as well as several changes in other secondary structures, leading to changes in the IR spectra.^27^ Therefore, the results of this study showed that drug complexation leads to the greater preservation of the protein structure. Moreover, the FTIR spectrum of the drug-polymer mixture showed no absence of functional peaks related to polymers, and their spectra were similar. There were no new peaks in the drug-polymer mixture or shifts in peaks related to polymers; therefore, there are no chemical interactions between the drug and polymers.^40^

### Release kinetics of the prepared rod-shaped implant

**Figure 10:**
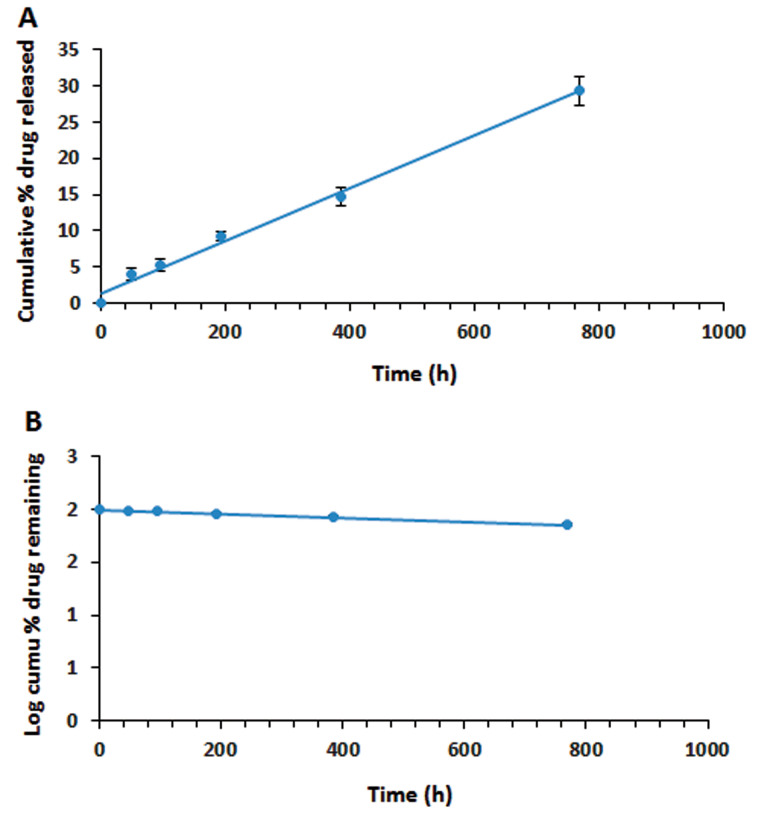
The release kinetics of ranibizumab (Fab): dextran sulfate sodium (DS) complex (Fab.com) as a protein drug: (A) zero-order model; and (B) first-order model which the error bars lie within the points (Values were represented as mean ± SD, n = 3).

The drug release kinetics and dissolution behavior of the implant, based on the *in vitro* analysis of drug release, were calculated as the cumulative percentage of the drug, the log cumulative percentage of the drug, and the log cumulative percentage of the remaining drug. Next, the curves were plotted, based on different kinetic parameters. To interpret the release kinetics, the R^2^ value was calculated, and then, comparisons were made to select the best kinetic model.^41^ Based on the calculated R^2^ values_,_ the kinetic model with the best fit was first-order model (R^2^=0.9928) for the ranibizumab implant, which showed a sustained drug release pertaining to its concentration. On the other hand, the calculated R^2^ value for zero-order kinetics was very close to the R^2^ value of the first-order kinetic model, showing the controlled-release formulation of the prepared implant (Figure 10).

In the first-order model, the release profile was dependent on the drug concentration in pharmaceutical formulations and could be used for dissolution of water-soluble ranibizumab in a porous matrix from the PHBV/PEG implant.^42^ Overall, PLGA is a very useful polymer for the sustained release of drugs and proteins due to its biocompatibility and biodegradability properties. The results of recent research on PLGA-based implants of ranibizumab indicated about 90% drug release within almost one month; also, the burst release of the drug was seen in these systems.^17,43^ However, in the present study, with application of PHBV polymer and drug complexation by DS, drug release occurred over a longer period; in other words, the drug was slowly released and controlled.

## Conclusion

The ranibizumab implant was successfully prepared by the melting method, with PHBV and PEG600 as pore formers. Also, *in vitro* characterization of the implant was carried out. The results showed that by complexation, the protein activity could be largely retained in melting conditions and that this method could be used for the development of implants, containing protein drugs, using an extruder in both research and industry. Also, the PHBV polymer could be used for preparing a sustained-release implant, containing protein drugs, such as ranibizumab. Because of its controlled release properties, it can be concluded that the ranibizumab implant is a promising option for diabetic retinopathy and other eye diseases associated with blindness. Overall, optimization of formulations, as well as *in vivo* research, is required for the development of effective dosage forms of this implant.

## Ethical Issues

Not applicable.

## Conflict of Interest

The authors declare no conflict of interest regarding the publication of this article.

## Acknowledgments

This study was granted by the Deputy of Research of Tehran University of Medical Sciences (grant number: 35574-30-03-96) and Iran National Science Foundation (grant number: 96004866). The authors would like to thank these institutions for their financial support.
